# The RNF8 and RNF168 Ubiquitin Ligases Regulate Pro- and Anti-Resection Activities at Broken DNA Ends During Non-Homologous End Joining

**DOI:** 10.1016/j.dnarep.2021.103217

**Published:** 2021-09-01

**Authors:** Bo-Ruei Chen, Yinan Wang, Zih-Jie Shen, Amelia Bennett, Issa Hindi, Jessica K. Tyler, Barry P. Sleckman

**Affiliations:** a Division of Hematology and Oncology, Department of Medicine, University of Alabama at Birmingham, Birmingham, AL, 35233, United States; b O’Neal Comprehensive Cancer Center, University of Alabama at Birmingham, Birmingham, AL, 35233, United States; c Department of Pathology and Laboratory Medicine, Weill Cornell Medicine, New York, NY, 10065, United States

**Keywords:** V(D)J recombination, NHEJ, DNA end resection, XLF, RNF8, RNF168

## Abstract

The RING-type E3 ubiquitin ligases RNF8 and RNF168 recruit DNA damage response (DDR) factors to chromatin flanking DNA double strand breaks (DSBs) including 53BP1, which protects DNA ends from resection during DNA DSB repair by non-homologous end joining (NHEJ). Deficiency of RNF8 or RNF168 does not lead to demonstrable NHEJ defects, but like deficiency of 53BP1, the combined deficiency of XLF and RNF8 or RNF168 leads to diminished NHEJ in lymphocytes arrested in G_0_/G_1_ phase. The function of RNF8 in NHEJ depends on its E3 ubiquitin ligase activity. Loss of RNF8 or RNF168 in G_0_/G_1_-phase lymphocytes leads to the resection of broken DNA ends, demonstrating that RNF8 and RNF168 function to protect DNA ends from nucleases, pos sibly through the recruitment of 53BP1. However, the loss of 53BP1 leads to more severe resection than the loss of RNF8 or RNF168. Moreover, in 53BP1-deficient cells, the loss of RNF8 or RNF168 leads to diminished DNA end resection. We conclude that RNF8 and RNF168 regulate pathways that both prevent and promote DNA end resection in cells arrested in G_0_/G_1_ phase.

## 1. Introduction

DNA double strand breaks (DSBs) are repaired by either non-homologous end joining (NHEJ) or homologous recombination (HR) [1–3]. NHEJ functions in all phases of the cell cycle to rejoin broken DNA ends and is the main pathway of DSB repair in G_1_-phase cells and non-cycling G_0_ cells [2]. In contrast, HR functions in the S- and G_2_-phases of the cell cycle using the sister chromatid as a template for precise DNA DSB repair [1,3]. DNA repair by either pathway relies on the recruitment of DNA damage response (DDR) factors to chromatin at DSBs [1,4]. Phosphorylation of the histone variant H2AX, forming γ-H2AX, in chromatin flanking DNA DSBs is an early step in the DDR that leads to the recruitment of several DDR factors including the RNF8 and RNF168 RING-type E3 ubiquitin ligases [5–7]. RNF168 ubiquitylates H2A and H2AX, promoting the recruitment of additional DDR factors, including 53BP1 and BRCA1 [5,6,8–15]. 53BP1, along with downstream effectors RIF1 and the Shieldin complex, protects DNA ends from resection, which can lead to single stranded DNA (ssDNA) overhangs that antagonize repair by NHEJ [16–18]. In contrast, BRCA1 promotes DNA end resection, which is required for DNA DSB repair by HR [1,3]. How these activities are balanced to promote DSB repair by HR or NHEJ is not completely known.

The assembly of antigen receptor genes occurs in G_0_/G_1_-phase developing lymphocytes through V(D)J recombination, a reaction that is initiated when the RAG-1 and RAG-2 proteins, which together from the RAG endonuclease, introduce DNA DSBs at the border of two recombining gene segments [19]. DNA cleavage by RAG leads to the formation of a hairpin sealed coding end (CE) and a blunt signal end (SE) at each DSB. The two CEs are then joined to form a coding join (CJ) and the two SEs joined to form a signal join (SJ) via NHEJ [20]. NHEJ is dependent on a set of core factors, including KU70, KU80, DNA Ligase IV and XRCC4, with loss of any of these factors leading to a complete block in NHEJ and RAG DSB repair [2,20]. There are additional NHEJ proteins that are not absolutely required for DSB repair. One of these, the XRCC4-like factor (XLF), functions during NHEJ in non-lymphoid cells but is largely dispensable for NHEJ in lymphoid cells [21–24]. However, lymphocytes with combined deficiencies of XLF and other DDR proteins have shown demonstrable defects in the repair of RAG DSBs during V(D) J recombination and thus revealed the functions of XLF and these proteins in NHEJ-mediated DSB repair [25–33]. In this regard, while loss of either XLF or 53BP1 leads to only modest defects in RAG DSB repair in lymphocytes, the combined loss of XLF and 53BP1 leads to a dramatic block in the NHEJ-mediated repair of these DSBs [27].

Here we examine the function of RNF8 and RNF168 during NHEJ in wild type and XLF-deficient lymphocytes. These analyses reveal that both RNF8 and RNF168 function during NHEJ. Moreover, we show that, like 53BP1, RNF8 and RNF168 protect DNA ends from nucleolytic resection in G_0_/G_1_-phase cells. However, DNA end resection in 53BP1-deficient cells is more severe than in RNF8-or RNF168-deficient cells and cells with a combined deficiency of 53BP1 and either RNF8 or RNF168 exhibit diminished resection as compared to cells only deficient in 53BP1. Thus, RNF8 and RNF168 function to balance pro- and anti-resection activities at broken DNA ends in G_0_/G_1_-phase cells.

## 2. Materials and Methods

### 2.1. Cell line generation and cells culture

Abelson virus-transformed pre-B cells (abl pre-B cells) were generated from the bone marrow of *DNA Lig4*^*LoxP/LoxP*^, *Xlf^−/−^*, *Rnf8*^*−/−*^ and *Xlf*^*−/−*^*: Rnf8^−/−^* mice as previously described [23,34–36]. *DNA Lig4^−/−^* abl pre-B cells were generated by Cre-mediated deletion of the conditionally targeted alleles as previously described [37]. *Lig4^−/−^*:*Rnf8*^*−/−*^*, Lig4^−/−^*: *Rnf168^−/−^* and *Lig4^−/−^*:*53bp1^−/−^* abl pre-B cells were generated by CRISPR/Cas9-mediated inactivation of *Rnf8, Rnf168* or 53bp1 genes in *Lig4^−/−^* cells. To generate abl pre-B cells with doxycycline-inducible FLAG-Cas9, abl pre-B cells were transduced with pCW-Cas9 (addgene, 50661) lentivirus, selected with 2 μg/ml puromycin for 7–10 days and clones isolated by serial dilution. Individual clones were treated with 3 μg/ml doxycycline for 2 days and expression of FLAG-Cas9 verified by flow cytometry using anti-FLAG antibody after cell permeabilization. To inactivate genes by CRIRPS/Cas9, lentiviruses expressing guide RNAs (pKLV1, addgene, 50946) were used to transduce abl pre-B cells with inducible Cas9, followed by treatment with 3 μg/ml doxycycline for 7 days. The resulting bulk deleted cells were analyzed directly or subjected to serial dilution to isolate clonal cell lines. To analyze V(D)J recombination, the pMG-INV retroviral recombination substrate was used to transduce abl pre-B cells and clones with a single pMG-INV integrant were isolated. Cells were treated with 3 μM imatinib for 4 days and pMG-INV rearrangement assessed by flow cytometry and Southern blot as previously described [38].

### 2.2. Western blot analyses

Cell lysate was collected by resuspending and boiling cells in 1X LDS sample buffer (Thermo Fisher, NP0007) and resolved by either 3–8% NuPAGE Tris-Acetate (Thermo Fisher, EA03785BOX) or 4–12% Bis-Tris NuPAGE (Thermo Fisher, NP0336BOX) gels. Anti-HA antibody is from Covance (MMS-101 P). CtIP antibody is kindly provided by Richard Baer (Columbia University). 53BP1 antibody is from Bethyl Laboratories (A300–272A). RNF168 antibody is from EMD Millipore (ABE367). KAP1 antibody is from Genetex (GTX102226). GAPDH antibody is from Sigma (G8795).

### 2.3. Southern blot analyses

10 μg of genomic DNA from abl pre-B cells with pMG-INV was digested with *NheI* or *XbaI* and resolved in 1.2% TAE gels. Upon transferring DNA to Zeta Probe (Bio Rad, 1620165) membranes, V(D)J recombination products and intermediates of pMG-INV were visualized using Thy1 probe (800 bp Thy1.1 cDNA) as previously described [26]. Quantification of the intact SEs in *Lig4^−/−^* and other cell lines derived from *Lig4*^*−/−*^ was done using Image J and calculated as the percentage of the intensity of intact signal ends (iSEs) over the total intensity of unrearranged substrate (UR), iSEs and resected signal ends (rSE) (the region from 0.5 to 3.7 kb). Southern blot analyses were carried out on two independently isolated mutants of each genotype for at least twice.

### 2.4. Retroviral expression of WT and mutant RNF8

The murine RNF8 coding sequence was amplified from cDNA clone BC021778 (transOMIC) by PCR and cloned to pOZ-FH-N downstream of FLAG-HA epitope tag at *XhoI* and *NotI* sites. DNA sequences corresponding to amino acids 37–109 and 405–447 are deleted in mutant RNF8 (ΔFHA) and RNF8 (ΔRING), respectively.

### 2.5. Flow cytometric analysis of radiation-induced chromatin association of RPA

Analysis of DNA-damage induced RPA binding to chromatin was performed as previously described with modifications [39]. Briefly, abl pre-B cells were treated with 3 μM imatinib for 2 days, followed by 10 Gy irradiation. 8 hours after irradiation, cells were permeabilized in 150 μl of 0.05% Triton X-100/1X PBS on ice for 10 min and immediately diluted in 2 ml of FACS wash (2% fetal bovine serum (FBS)/1X PBS). Permeabilized cells were fixed in 150 μl of BD Cytofix/Cytoperm at room temperature for 15 min and then washed in 1 ml of FACS wash. Cells were then stained with anti-RPA antibody (Cell Signaling, 2208S) and Alexa Fluro 647 anti-rat IgG (Biolegend, 405416) and analyzed with an LSRII (BD Biosciences). Two independently isolated mutants of each genotype were used in the flow cytometric assays and repeated at least twice.

## 3. Results

### 3.1. RNF8 functions during NHEJ

**Fig. 1.**
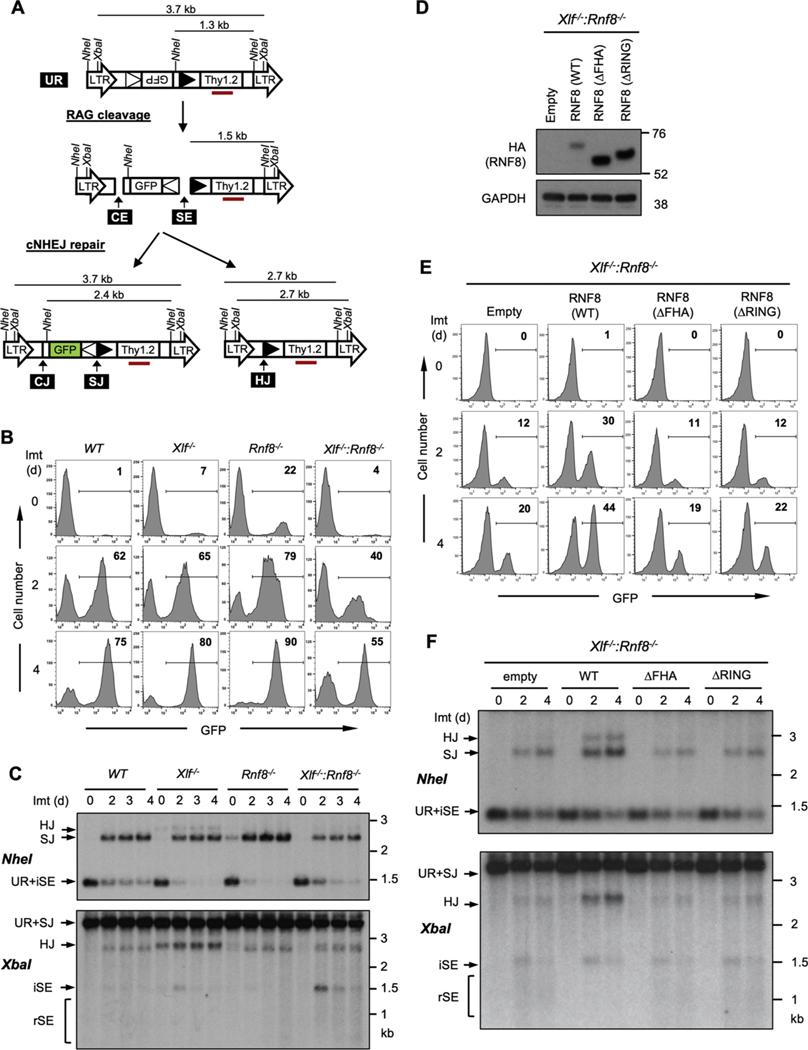
Combined deficiency of XLF and RNF8 impairs NHEJ during V(D)J recombination. (A) Schematic of the unrearranged V (D)J recombination substrate pMG-INV (UR), the signal end (SE) and coding end (CE) intermediates, and the resulting signal join (SJ), coding join (CJ) and hybrid join (HJ). The longterminal repeats (LTR), *NheI* and *XbaI* restriction sites, recombination signal sequences (open and filled triangles), GFP cDNA, Thy1.2 cDNA, and Thy1 probe (red rectangle) are indicated. (B) Flow cytometric analysis of GFP expression from pMG-INV in the indicated abl pre-B cells after imatinib treatment for the indicated times. The numbers in the top-right corners of histograms indicate the percentage of GFP-expressing cells. Imt (d) = days in imatinib. (C) Southern blot analysis of genomic DNA from cells in panel B after digestion with *NheI* or *XbaI* and probed with the Thy1 probe (panel A). iSE = intact signal end. rSE = resected signal end. (D) Western blot of whole cell lysates with anti-HA antibody to detect WT RNF8 and the FHA (ΔFHA) and RING (ΔRING) domain RNF8 mutants. (E) Flow cytometric analysis of imatinib-treated *Xlf*^*−/−*^: *Rnf8^−/−^* abl pre-B cells and those expressing WT RNF8 or the ΔFHA or ΔRING mutants as described in Fig. 1B. (F) Southern blot analysis of genomic DNA from cells from panel E as described in Fig. 1C.

Murine Abelson virus-transformed pre-B cells (abl pre-B cells) were generated containing a single copy of the pMG-INV retroviral recombination substrate (Fig. 1A) [26]. pMG-INV has two recombination signal sequences (RSs) flanking an antisense GFP cDNA (Fig. 1A) [26]. RAG cleavage at the pMG-INV RSs leads to the generation of a pair of signal ends (SEs) and coding ends (CEs) at each DSB (Fig. 1A). NHEJ then joins the two SEs to generate a signal join (SJ) and the two CEs to generate a coding join (CJ) leading to inversion of the GFP cDNA and GFP expression as an indicator of completed V(D)J recombination (Fig. 1A). The CE from one DSB can also be aberrantly joined to the SE from the other DSB to generate a hybrid join (HJ) (Fig. 1A). To induce V(D)J recombination, abl pre-B cells are treated with the abl kinase inhibitor, imatinib, which leads to G_0_/G_1_ cell cycle arrest, induction of RAG and V (D)J recombination at pMG-INV [36] as indicated by robust GFP expression in WT cells (Fig. 1B).

RNF8-deficident abl pre-B cells were generated from *Rnf8*^*−/−*^ mice and pMG-INV was introduced into these cells to monitor V(D)J recombination and NHEJ [34] and treatment with imatinib leads to pMG-INV recombination equivalent to WT abl pre-B cells as indicated by GFP expression (Fig. 1B). Similar to what was observed in WT abl pre-B cells, Southern blot analyses revealed pMG-INV SJ formation with no significant accumulation of un-repaired SEs at RAG DSBs in *Rnf8*^*−/−*^ abl pre-B cells (Fig. 1C). We conclude that RNF8 deficiency does not lead to a demonstrable defect in RAG DSB repair during V(D)J recombination in abl pre-B cells.

We next generated abl pre-B cells from *Xlf*^*−/−*^ and *Xlf*^*−/−*^*:Rnf8*^*−/−*^ mice, followed by integration of the pMG-INV reporter. *Xlf*^*−/−*^ abl pre-B cells exhibit robust V(D)J recombination of pMG-INV, similar to WT abl pre-B cells as evidenced by GFP expression (Fig. 1B) and Southern blot analyses of SJ formation (Fig. 1C) [23]. In contrast, *Xlf*^*−/−*^*:Rnf8*^*−/−*^ abl pre-B cells exhibited a decrease in the fraction of GFP expressing cells after RAG induction, indicative of a defect in V(D)J recombination (Fig. 1B). Indeed, there was a reduction in the hybridization intensity of the SJ band in *Xlf*^*−/−*^*:Rnf8*^*−/−*^ abl pre-B cells as compared to the *WT*, *Xlf*^*−/−*^ and *Rnf8*^*−/−*^ abl pre-B cells (Fig. 1C). Moreover, un-repaired SEs were detectable by Southern blot in *Xlf*^*−/−*^*:Rnf8*^*−/−*^ abl pre-B cells (Fig. 1C). Notably, the intensity of these SE bands diminishes over time, possibly reflecting the nucleolytic resection of the DNA ends (see below).

There are two conserved domains in RNF8, the FHA and RING domains [5,11]. The FHA domain promotes the association of RNF8 with phosphorylated MDC-1 at the site of a DSB and the RING domain is required for RNF8 E3 ligase catalytic activity [11,12]. To determine if these domains of RNF8 are required for NHEJ we expressed wild type RNF8 or mutants lacking the FHA (ΔFHA) or RING (ΔRING) domains in *Xlf*^*−/−*^*:Rnf8*^*−/−*^ abl pre-B cells (Fig. 1D). As compared to cells infected with empty vector, induction of V(D)J recombination in *Xlf*^*−/−*^*:Rnf8*^*−/−*^ abl pre-B cells expressing RNF8 resulted in a significant rescue of pMG-INV rearrangement as evidenced by GFP expression and Southern blot analyses (Fig. 1E and F). However, expression of either the ΔFHA or ΔRING RNF8 mutants, even though their levels were elevated over that of WT RNF8 (Fig. 1D), failed to improve pMG-INV rearrangement. We conclude that RNF8 functions during NHEJ-mediated DSB repair in ways that depend on the FHA and RING domains (Fig. 1E and F).

### 3.2. RNF8 and RNF168 are epistatic during NHEJ

**Fig. 2.**
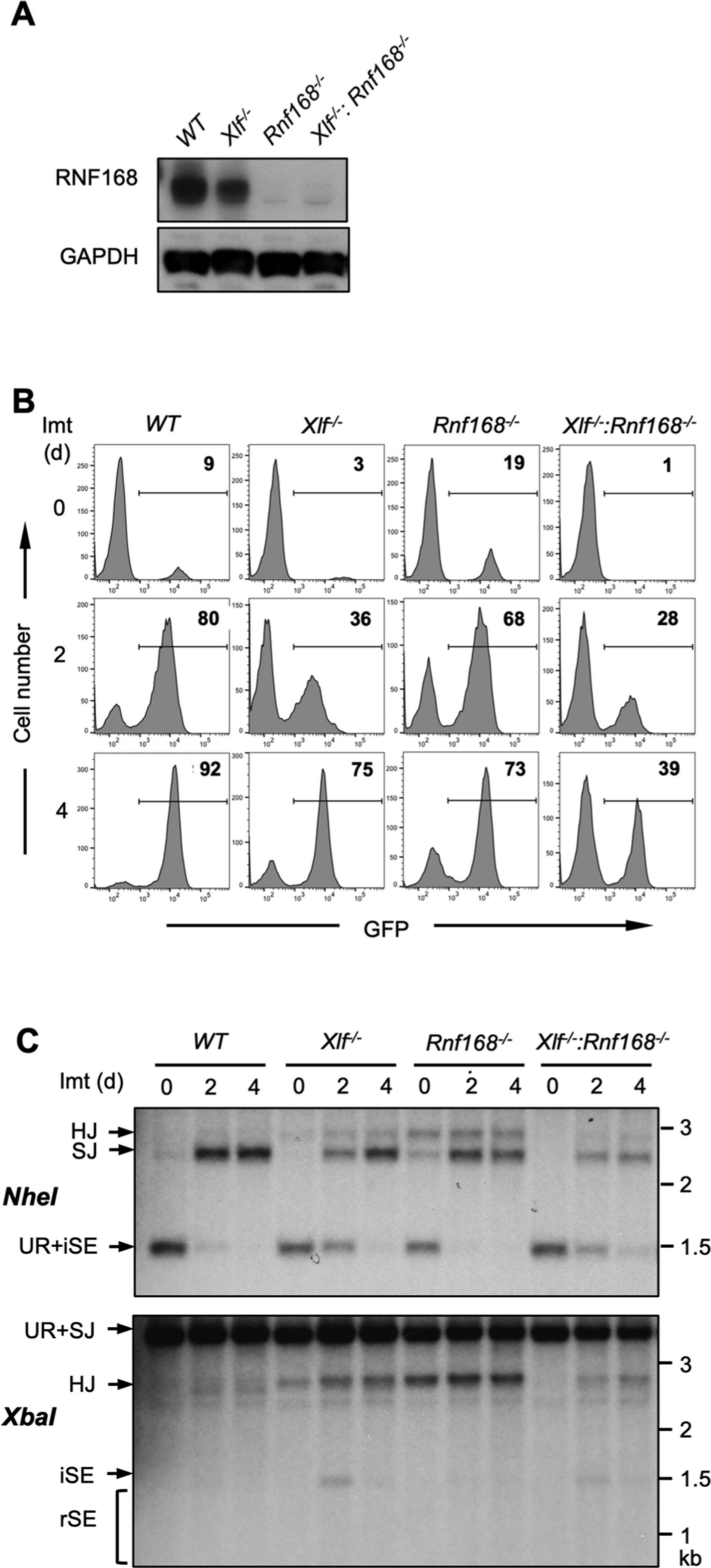
Combined deficiency of XLF and RNF168 impairs NHEJ during V (D)J recombination. **(**A) Western blot of whole cell lysates from the indicated cells probed with RNF168 and GAPDH antibodies. (B) Flow cytometric analysis of GFP expression from pMG-INV in the indicated abl pre-B cells after imatinib treatment for the indicated times. The numbers in the top-right corners of histograms indicate the percentage of GFP-expressing cells. (C) Southern blot analysis of genomic DNA from cells in panel B after digestion with *NheI* or *XbaI* and probed with the Thy1 probe as described in Fig. 1A.

RNF168 has functions downstream of RNF8 in response to DNA damage [6,8]. To determine if RNF168 also functions in NHEJ, *Rnf168*^*−/−*^ and *Xlf*^*−/−*^*:Rnf168*^*−/−*^ abl pre-B cells were generated from WT and *Xlf*^*−/−*^ abl pre-B cells through the CRISPR/Cas9-mediated inactivation of both *Rnf168* alleles and the loss of RNF168 protein in these cells was confirmed by Western blot (Fig. 2A). Similar to *Rnf8*^*−/−*^ abl pre-B cells, *Rnf168*^*−/−*^ abl pre-B cells had no detectable defect in V(D)J recombination (Fig. 2B and C). However, *Xlf*^*−/−*^*:Rnf168*^*−/−*^ abl pre-B cells have defects in NHEJ as evidenced by flow cytometric analyses of GFP expression and Southern blot analysis of SJ formation (Fig. 2B and C).

**Fig. 3.**
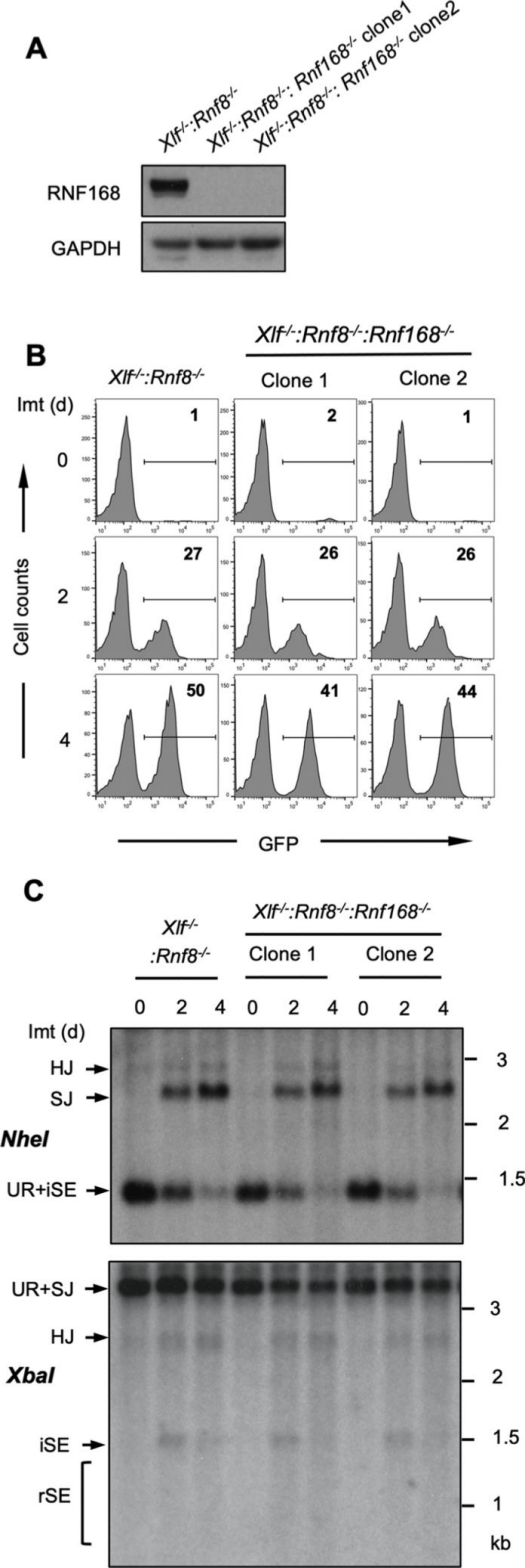
RNF8 and RNF168 have epistatic NHEJ functions. (A) Western blot of whole cell lysates from *Xlf−/−*:*Rnf8^−/−^* and *Xlf^−/−^*:*Rnf8^−/−^*:*Rnf168^−/−^* abl pre-B cells with RNF168 or GAPDH antibodies. (B) Flow cytometric analyses of GFP expression in imatinib-treated *Xlf*^*−/−*^*:Rnf8*^*−/−*^ and *Xlf^−/−^*:*Rnf8^−/−^*:*Rnf168^−/−^* abl pre-B cells after imatinib treatment for the indicated times. The numbers in the top-right corners of histograms indicate the percentage of GFP-expressing cells. (C) Southern blot analysis of genomic DNA from cells shown in panel B as in Fig. 1C.

To determine whether the functions of RNF8 and RNF168 are epistatic during NHEJ, we generated *Xlf*^*−/−*^*:Rnf8*^*−/−*^:*Rnf168*^*−/−*^ abl pre-B cells by introducing inactivating mutations at both *Rnf168* alleles by CRISPR/Cas9 in *Xlf*^*−/−*^*:Rnf8*^*−/−*^ abl pre-B cells (Fig. 3A). After imatinib treatment, *Xlf*^*−/−*^*:Rnf8*^*−/−*^*:Rnf168*^*−/−*^ abl pre-B cells exhibit GFP expression from pMG-INV rearrangement of similar magnitude to that observed in *Xlf*^*−/−*^*:Rnf8*^*−/−*^ abl pre-B cells (Fig. 3B). Moreover, Southern blot analysis revealed similar levels of RAG DSB repair and accumulation of un-repaired SEs in *Xlf*^*−/−*^*:Rnf8*^*−/−*^ and *Xlf*^*−/−*^*:Rnf8*^*−/−*^*:Rnf168*^*−/−*^ abl pre-B cells (Fig. 3C). We conclude that RNF8 and RNF168 function in the same pathway during NHEJ-mediated DSB repair.

### 3.3. RNF8 and RNF168 inhibit DNA end resection in G_0_/G_1_-phase cells

**Fig. 4.**
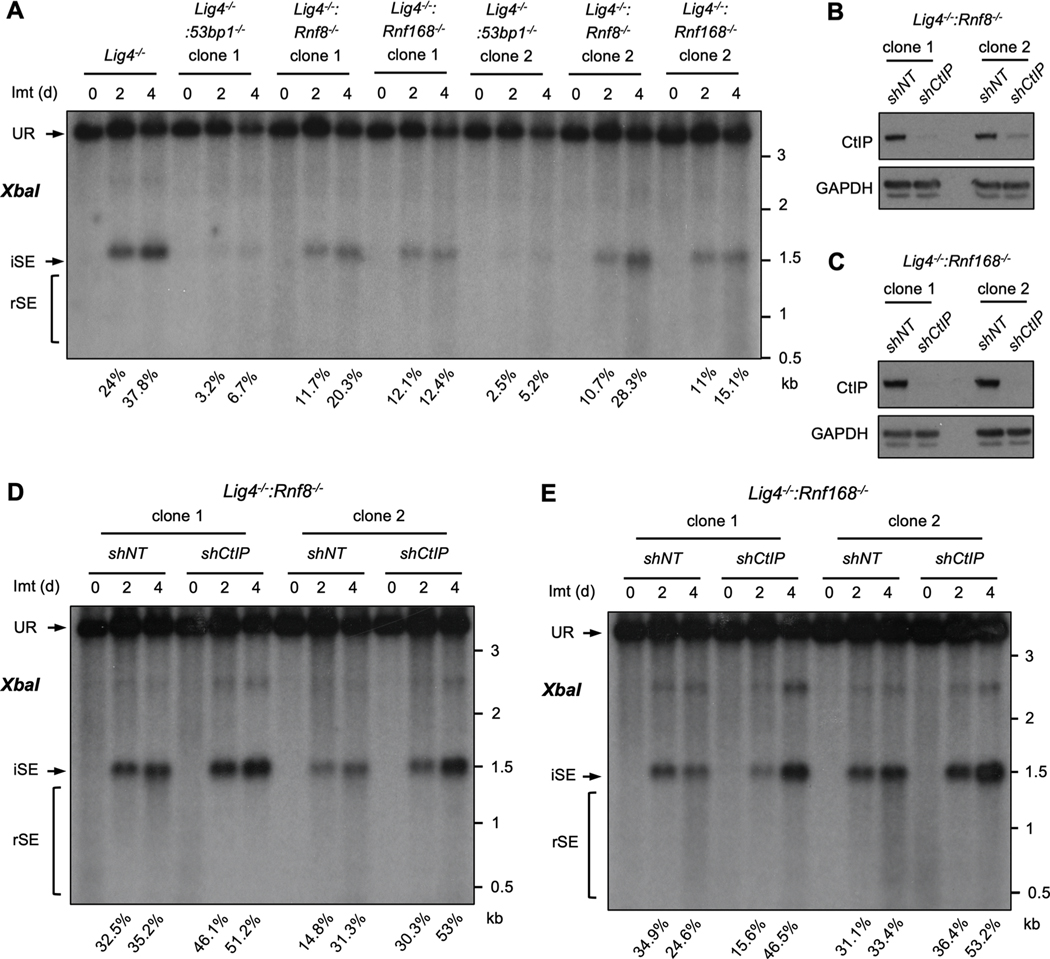
RNF8 and RNF168 protect DNA ends from resection. (A) Southern blot analysis of *XbaI*-digested genomic DNA from imatinib-treated *Lig4^−/−^*, *Lig4^−/−^*:*53bp1^−/−^*, *Lig4^−/−^*:*Rnf8^−/−^* or *Lig4^−/−^*:*Rnf168^−/−^* abl pre-B cells as described in Fig. 1C. Percentages of intact SEs (iSEs) in imatinib-treated samples are listed below each lane. (B, C) Western blot analyses of whole cell lysates from *Lig4^−/−^*:*Rnf8^−/−^* (B) or *Lig4^−/−^*:*Rnf168^−/−^* (C) abl pre-B cells expressing non-targeting (*shNT*) or CtIP (*shCtIP*) shRNAs using CtIP or GAPDH antibodies. (D, E) Southern blot analysis of *XbaI*-digested genomic DNA from *Lig4^−/−^*:*Rnf8^−/−^* or *Lig4^−/−^*:*Rnf168^−/−^* abl pre-B cells expressing *shNT* or *shCtIP* treated with imatinib for the indicated times as described in Fig. 1C. Percentages of intact SEs (iSEs) in imatinib-treated samples are listed below each lane.

RNF8 and RNF168 function to localize 53BP1 to DSBs and 53BP1 functions to protect DNA ends from resection [5,6,8,11,12,17]. To determine whether RNF8 and RNF168 function to protect DNA ends from resection in cells arrested in G_0_/G_1_ phase, we used abl pre-B cell lines deficient in DNA Ligase IV (*Lig4*^*−/−*^) to generate cells deficient in DNA Ligase 4 and 53BP1 (*Lig4*^*−/−*^:*53bp1*^*−/−*^), RNF8 (*Lig4*^*−/−*^:*Rnf8*^*−/−*^) or RNF168 (*Lig4*^*−/−*^:*Rnf168*^*−/−*^) by CRISPR/Cas9 inactivation of both *53bp1*, *Rnf8* or *Rnf168* alleles, respectively (Fig. S1). Treatment of *Lig4*^*−/−*^ abl pre-B cells with imatinib led to RAG cleavage at pMG-INV, and RAG DSBs persist un-repaired due to the deficiency of DNA Ligase 4 and the lack of NHEJ in these cells (SE, Fig. 4A). The persistence of DSBs in these cells allows to better analyze DNA end processing in response to different genetic manipulations. The hybridizing band due to the un-repaired SEs in *Lig4*^*−/−*^ abl pre-B cells remained homogenous in size over four days indicating that these DNA ends were not significantly resected (Fig. 4A). In contrast, the hybridizing bands from SEs generated in *Lig4*^*−/−*^:*53bp1*^*−/−*^ abl pre-B cells were significantly reduced in intensity, shown as the reduced percentage of intact SEs (iSEs) in these cells when compared with that in *Lig4*^*−/−*^ abl pre-B cells, and existed as a heterogeneously sized ‘smear’ of lower molecular weight bands indicative of extensive DNA end resection (rSEs) (Fig. 4A) [40,41].

The proportion of intact, unprocessed SEs in *Lig4*^*−/−*^:*Rnf8*^*−/−*^ and *Lig4*^*−/−*^:*Rnf168*^*−/−*^ abl pre-B cells is also lower than that in *Lig4*^*−/−*^ abl pre-B cells and heterogenous bands of small sizes could also be detected as *Lig4*^*−/−*^:*53bp1*^*−/−*^ abl pre-B cells, suggesting the SEs in these cells also exhibited resection, although to a much lesser extent than what was observed in *Lig4*^*−/−*^:*53bp1*^*−/−*^ abl pre-B cells (Fig. 4A). That these DNA ends were resected was further evidenced by the shRNA-mediated knockdown of CtIP, which is required for the resection of RAG DSBs in G_0_/G_1_-arrested abl pre-B cells (Fig. 4B and C)[37]. Reduced expression of CtIP in *Lig4*^*−/−*^:*Rnf8*^*−/−*^ and *Lig4*^*−/−*^:*Rnf168*^*−/−*^ abl pre-B cells led to an increased intensity of the full length SE band and a reduction in the lower molecular weight smear, indicative of diminished resection of broken DNA ends (Fig. 4D and E).

**Fig. 5.**
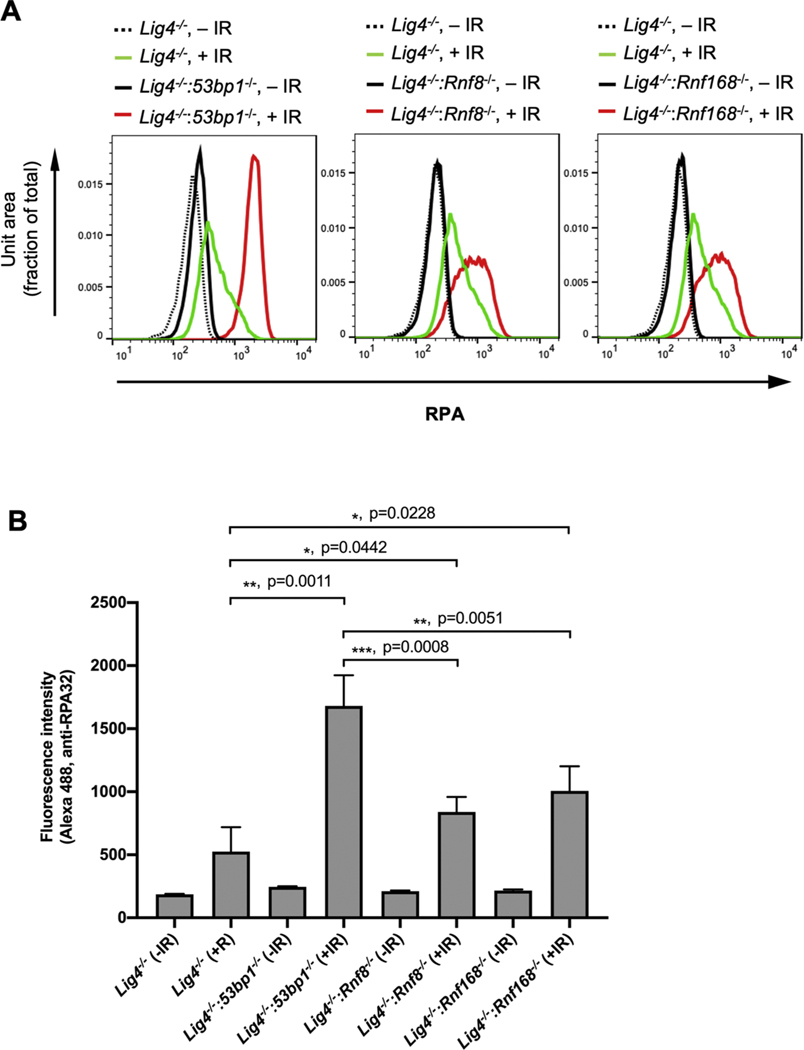
Deficiency in RNF8 or RNF168 promotes DNA damage-induced RPA binding to chromatin. (A) Flow cytometric analyses chromatin-bound RPA using RPA32 antibody after permeabilization of un-irradiated (dotted and solid black lines) or irradiated (10 Gy, green or red lines) imatinib-treated *Lig4^−/−^*, *Lig4*^*−/−*^*:53bp1*^*−/*^−, *Lig4*^*−/−*^*: Rnf8*^*−/−*^ or *Lig4*^*−/−*^*: Rnf168*^*−/−*^ abl pre-B cells. The experiments were carried out on two independently generated *Lig4*^*−/−*^*:53bp1*^*−/−*^, *Lig4*^*−/−*^*: Rnf8*^*−/−*^ or *Lig4*^*−/−*^*: Rnf168*^*−/*^*−* abl pre-B cells. Representative histograms are shown. (B) Mean ± SD of median intensities of chromatin-bound RPA staining in RPA flow cytometric analyses shown in (A). Unpaired t-test was used for statistic analysis. n = 4 for all cell lines except *Lig4^−/−^* (n = 3).

To assess whether the role of RNF8- and RNF168 in limiting DNA end resection extended beyond V(D)J recombination, we irradiated *Lig4*^*−/−*^, *Lig4*^*−/−*^:*53bp1*^*−/−*^, *Lig4*^*−/−*^:*Rnf8*^*−/−*^ and *Lig4*^*−/−*^:*Rnf168*^*−/−*^ abl pre-B cells that had been arrested in G_0_/G_1_ by treatment with imatinib and assayed for RPA association with chromatin by flow cytometry (Fig. 5) [39]. The RPA complex associates with single strand DNA (ssDNA) generated by nucleolytic resection at broken DNA ends and therefore the level of RPA associated with chromatin after DNA damage is indicative of ssDNA formation at DSBs [1]. After irradiation, *Lig4*^*−/−*^ abl pre-B cells exhibited much lower levels of chromatin-bound RPA when compared to *Lig4*^*−/−*^:*53bp1*^*−/−*^ abl pre-B cells, consistent with the function of 53BP1 in protecting broken DNA ends from nuclease activity [16–18,41]. Irradiated G_0_/G_1_-arrested *Lig4*^*−/−*^:*Rnf8*^*−/−*^ and *Lig4*^*−/−*^:*Rnf168*^*−/−*^ abl pre-B cells also exhibited increased RPA association with chromatin indicative of the formation of ssDNA at irradiation-induced DSBs (Fig. 5). There was less chromatin-bound RPA in irradiated *Lig4*^*−/−*^ abl pre-B cells lacking RNF8 or RNF168 as compared to those lacking 53BP1, suggesting that there is more ssDNA at DSBs in cells lacking 53BP1 as compared to those lacking RNF8 or RNF168 (Figs. 4A and 5). We conclude that RNF8 and RNF168 have functions that can inhibit DNA end resection at DSBs in G_0_/G_1_-phase cells at both RAG generated and irradiation induced DNA ends.

### 3.4. RNF8 and RNF168 promote DNA end resection in G_0_/G_1_-phase cells

**Fig. 6.**
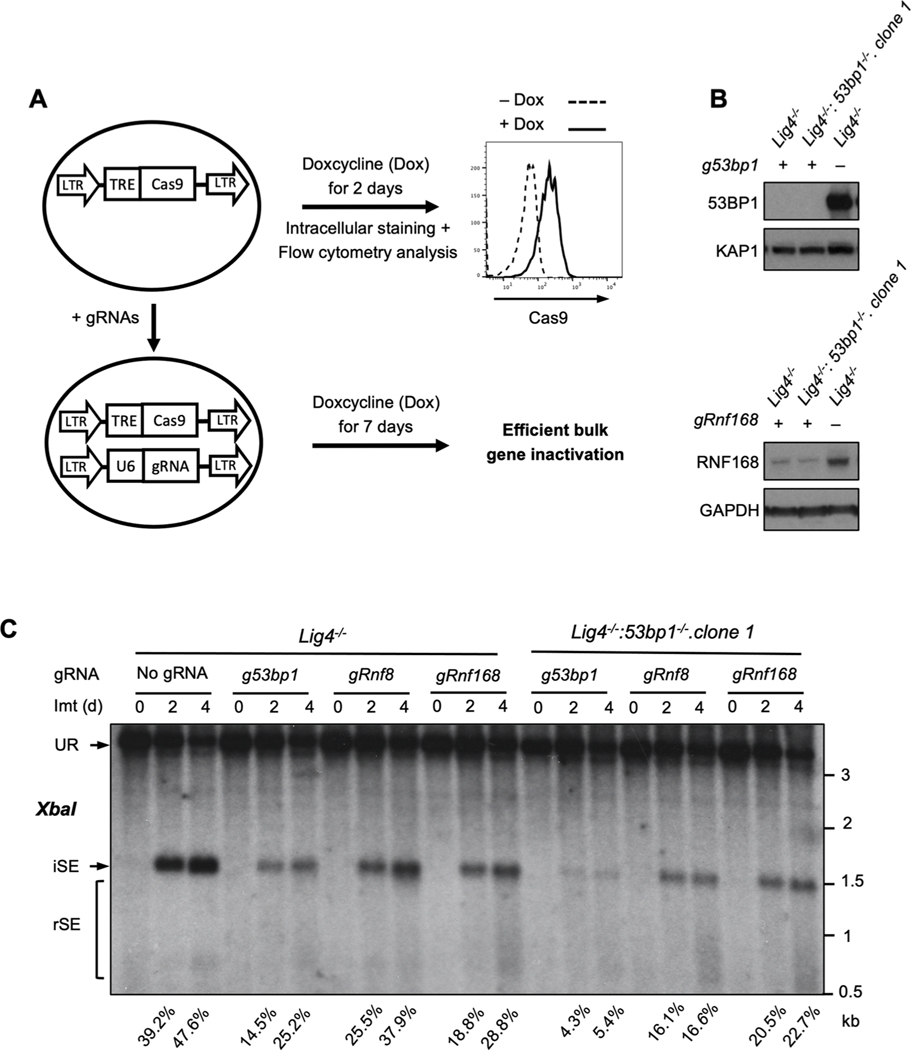
RNF8 and RNF168 promote resection in *Lig4^−/−^*:*53bp1^−/−^* cells. (A) Schematic of tetracycline-inducible CRISPR/Cas9-mediated gene inactivation in bulk abl pre-B cell populations using guide RNAs (gRNAs). Histogram of the flow cytometric analysis of FLAG-tagged Cas9 using FLAG antibody on permeabilized abl pre-B cells before (dashed line) and after (solid line) doxycycline (Dox) induction is also shown. (B) Western blot analyses with 53BP1 or RNF168 antibodies on cell lysate from *Lig4^−/−^* or *Lig4^−/−^:53bp1^−/−^* abl pre-B cells with chromosomally integrated tetracycline-inducible Cas9, expressing gRNAs targeting *53bp1* (*g53bp1*) or *Rnf168* (*gRnf168*). (C) Southern blot analysis as described in Fig. 1C of *XbaI*-digested genomic DNA from imatinib-treated *Lig4^−/−^* or *Lig4^−/−^:53bp1^−/−^* abl pre-B cells with chromosomally integrated tetracycline-inducible Cas9, expressing guide RNAs targeting *53bp1* (*g53bp1*), *Rnf8* (*gRnf8*) or *Rnf168* (*gRnf168*) after 7 days of doxycycline treatment for gene inactivation. Percentages of intact SEs (iSEs) in imatinib-treated samples are listed below each lane.

RNF8 and RNF168 promote the association of 53BP1 with DSBs, yet we find less resection of DSBs (higher percentage of intact SEs) in G_0_/G_1_-phase cells deficient in RNF8 or RNF168 as compared to those deficient in 53BP1 (Figs. 4A and 5). This suggests that RNF8 and RNF168 may also have functions in promoting resection. To test this, we developed a CRISPR/Cas9 approach to efficiently inactivate both alleles of *53bp1*, *Rnf8* or *Rnf168* in bulk populations of *Lig4*^*−/−*^ and *Lig4*^*−/−*^*:53bp1*^*−/−*^ abl pre-B cells (Fig. 6A). A lentivirus encoding doxycycline inducible Cas9 was introduced into *Lig4−*^*/−*^ or *Lig4*^*−/−*^*:53bp1*^*−/−*^ abl pre-B cells followed by introduction of lentiviruses expressing guide RNAs (gRNAs) targeting *53bp1*, *Rnf8* or *Rnf168*. Treatment of these cells with doxycycline for 7 days led to significant loss of 53BP1 and RNF168 protein in the bulk populations, a process we termed bulk gene inactivation (Fig. 6A and B). Similar to established clonal *Lig4*^*−/−*^*:53bp1*^*−/−*^, *Lig4*^*−/−*^:*Rnf8*^*−/−*^ or *Lig4*^*−/−*^: *Rnf168*^*−/−*^ abl pre-B cells, bulk deletion of *53bp1*, *Rnf8* or *Rnf168* in *Lig4*^*−/−*^ abl pre-B cells led to resection of un-repaired SEs (Fig. 6C). Remarkably, bulk deletion of *Rnf8* or *Rnf168* alleles in *Lig4*^*−/−*^*:53bp1*^*−/−*^ abl pre-B cells led to a reduction in the resection of SEs (increased proportion of intact SEs) (Figs. 6C and S2). Thus, in G_0_/G_1_-phase *Lig4*^*−/−*^*:53bp1*^*−/−*^ abl pre-B cells, RNF8 and RNF168 activity is required to promote DNA end resection. Together, our findings indicate that RNF8 and RNF168 have both pro- and anti-resection activities at DSBs in G_0_/G_1_-phase cells.

## 4. Discussion

Here we show that the RNF8 and RNF168 RING E3 ubiquitin ligases function in NHEJ-mediated DSB repair regulating DNA end resection during V(D)J recombination and after irradiation in lymphocytes. Although RNF8 or RNF168 deficiency alone does not lead to a defect in V(D)J recombination, the combined deficiency of either of these proteins with XLF leads to a demonstrable defect in RAG DSB repair. Therefore, while RNF8 and RNF168 are not required for NHEJ under normal conditions, their roles in NHEJ can be revealed in the absence of XLF through a “synthetic NHEJ defect” resulting from combining the deficiency of non-essential functions of these proteins. The function of RNF8 in NHEJ requires both the FHA and RING domains suggesting that both the localization of RNF8 to DSBs through phospho-MDC1 (FHA domain) and its E3 ubiquitin ligase activity (RING domain) are important in this context. Moreover, our finding that these two proteins are epistatic with respect to their activities in NHEJ-mediated DSB repair is consistent with the requirement for RNF8 to recruit RNF168 to DSBs and raises the possibility that the primary role of RNF8 is in recruiting RNF168 [6,8].

How is it that RNF8 and RNF168 function during NHEJ? One explanation may be that they protect DNA ends until they can be joined by NHEJ. We find that the loss of RNF8 or RNF168 can lead to the resection of DNA ends generated by RAG cleavage in G_0_/G_1_-phase abl pre-B cells that are unable to repair DSBs due to deficiency of DNA Ligase 4. Moreover, irradiation of G_0_/G_1_-phase abl pre-B cells that are deficient in DNA Ligase 4 and either RNF8 or RNF168 leads to increased association of RPA with chromatin indicative of ssDNA formation at DSBs [2,42]. The formation of these ssDNA overhangs would antagonize efficient NHEJ-mediated DSB repair [2,42]. Under normal circumstances where NHEJ rapidly repairs DSBs, the activity of RNF8 and RNF168 in protecting DNA ends from resection may not be required as the broken ends are joined before they can be resected. However, when DNA end joining kinetics is slowed, as may be the case in cells deficient in XLF, which bridges and stabilizes DNA ends, there may be a greater reliance on pathways that protect DNA ends from resection until they can be joined [43–46].

In response to DNA DSBs, H2AX is phosphorylated to form γH2AX that subsequently promotes the association of RNF8, RNF168 and 53BP1 with chromatin flanking broken DNA ends and these proteins maintain the integrity of DNA ends [5,6,8,11,12,37,47–49]. While neither H2AX nor 53BP1 deficiency alone leads to demonstrable NHEJ defects in lymphocytes, the combined deficiency of H2AX or 53BP1 and XLF leads to an NHEJ defect reminiscent of the NHEJ defect we observed upon depletion of RNF8 or RNF168 in XLF-deficient abl pre-B cells [27,29]. Thus, the NHEJ defect observed in cells with combined deficiencies in XLF and H2AX, 53BP1, RNF8 or RNF168 could reflect delayed joining kinetics (due to XLF deficiency) combined with defective DNA end protection (due to H2AX, 53BP1, RNF8 or RNF168 deficiency).

RNF8 and RNF168 function to recruit 53BP1 to chromatin at DNA DSBs in a manner that depends on their E3 ubiquitin ligase activities [6, 8,11,12,15]. Surprisingly, we found that in G_0_/G_1_-phase lymphocytes, the extent of DNA end resection in cells deficient in RNF8 or RNF168 was much less than that observed in cells deficient in 53BP1. Moreover, we find that DNA end resection is diminished in 53BP1-deficient cells that are also deficient in RNF8 or RNF168. This suggests that while RNF8 and RNF168 function to protect DNA ends, they also regulate activities that promote DNA end resection in G_0_/G_1_-phase cells. Given that RNF8 and RNF168 recruit BRCA1 to DNA DSBs, it is conceivable that they could also recruit proteins that function to promote DNA end resection, such as nucleases, in G_0_/G_1_-phase cells [6,8,11,12]. Moreover, the ubiquitylation of histones in chromatin at DSBs by RNF8 or RNF168 may alter chromatin structure in a manner that makes broken DNA ends more accessible to the resection machinery [4,50].

While DNA end resection is essential for homologous recombination in S/G_2_-phase of the cell cycle, processing of DNA ends is more limited when cells enter the G_0_ or G_1_ phase of the cell cycle where they rely on NHEJ for efficient DSB repair [2,37,42,51,52]. Extensive DNA end resection in G_0_/G_1_-phase cells would limit NHEJ and promote aberrant homology-mediated joining of DNA ends with ssDNA overhangs leading to genome instability [2,42]. However, during NHEJ in G_0_/G_1_-phase cells, some DNA end resection can be required to form compatible DNA ends for joining and for the processing of DNA ends with structures that would otherwise prohibit joining by NHEJ [2,42]. Therefore, in G_0_/G_1_-phase cells, pathways must exist that regulate DNA end resection activities to favor DNA end processing that promotes NHEJ. We propose that RNF8 and RNF168 function to balance pro- and anti-resection activities to provide optimal processing of DNA ends for joining during NHEJ.

## Supplementary Material

Figure S1

Figure S2

## References

[1] Ciccia A, Elledge SJ, The DNA damage response: making it safe to play with knives, Mol Cell 40 (2010) 179–204. 20965415 10.1016/j.molcel.2010.09.019 PMC2988877

[2] Chang HHY, Pannunzio NR, Adachi N, Lieber MR, Non-homologous DNA end joining and alternative pathways to double-strand break repair, Nat Rev Mol Cell Biol 18 (2017) 495–506. 28512351 10.1038/nrm.2017.48 PMC7062608

[3] Prakash R, Zhang Y, Feng W, Jasin M, Homologous recombination and human health: the roles of BRCA1, BRCA2, and associated proteins, Cold Spring Harb Perspect Biol 7 (2015), a016600. 10.1101/cshperspect.a016600 PMC4382744 25833843

[4] Price BD, D’Andrea AD, Chromatin remodeling at DNA double-strand breaks, Cell 152 (2013) 1344–1354. 23498941 10.1016/j.cell.2013.02.011 PMC3670600

[5] Kolas NK, Chapman JR, Nakada S, Ylanko J, Chahwan R, Sweeney FD, Panier S, Mendez M, Wildenhain J, Thomson TM, Pelletier L, Jackson SP, Durocher D, Orchestration of the DNA-damage response by the RNF8 ubiquitin ligase, Science 318 (2007) 1637–1640. 18006705 10.1126/science.1150034 PMC2430610

[6] Doil C, Mailand N, Bekker-Jensen S, Menard P, Larsen DH, Pepperkok R, Ellenberg J, Panier S, Durocher D, Bartek J, Lukas J, Lukas C, RNF168 binds and amplifies ubiquitin conjugates on damaged chromosomes to allow accumulation of repair proteins, Cell 136 (2009) 435–446. 19203579 10.1016/j.cell.2008.12.041

[7] Fernandez-Capetillo O, Lee A, Nussenzweig M, Nussenzweig A, H2AX: the histone guardian of the genome, DNA Repair (Amst) 3 (2004) 959–967. 15279782 10.1016/j.dnarep.2004.03.024

[8] Stewart GS, Panier S, Townsend K, Al-Hakim AK, Kolas NK, Miller ES, Nakada S, Ylanko J, Olivarius S, Mendez M, Oldreive C, Wildenhain J, Tagliaferro A, Pelletier L, Taubenheim N, Durandy A, Byrd PJ, Stankovic T, Taylor AM, Durocher D, The RIDDLE syndrome protein mediates a ubiquitin-dependent signaling cascade at sites of DNA damage, Cell 136 (2009) 420–434. 19203578 10.1016/j.cell.2008.12.042

[9] Goldberg M, Stucki M, Falck J, D’Amours D, Rahman D, Pappin D, Bartek J, Jackson SP, MDC1 is required for the intra-S-phase DNA damage checkpoint, Nature 421 (2003) 952–956. 12607003 10.1038/nature01445

[10] Stewart GS, Wang B, Bignell CR, Taylor AM, Elledge SJ, MDC1 is a mediator of the mammalian DNA damage checkpoint, Nature 421 (2003) 961–966. 12607005 10.1038/nature01446

[11] Huen MS, Grant R, Manke I, Minn K, Yu X, Yaffe MB, Chen J, RNF8 transduces the DNA-damage signal via histone ubiquitylation and checkpoint protein assembly, Cell 131 (2007) 901–914. 18001825 10.1016/j.cell.2007.09.041 PMC2149842

[12] Mailand N, Bekker-Jensen S, Faustrup H, Melander F, Bartek J, Lukas C, Lukas J, RNF8 ubiquitylates histones at DNA double-strand breaks and promotes assembly of repair proteins, Cell 131 (2007) 887–900. 18001824 10.1016/j.cell.2007.09.040

[13] Wang B, Elledge SJ, Ubc13/Rnf8 ubiquitin ligases control foci formation of the Rap80/Abraxas/Brca1/Brcc36 complex in response to DNA damage, Proc Natl Acad Sci U S A 104 (2007) 20759–20763. 18077395 10.1073/pnas.0710061104 PMC2410075

[14] Sobhian B, Shao G, Lilli DR, Culhane AC, Moreau LA, Xia B, Livingston DM, Greenberg RA, RAP80 targets BRCA1 to specific ubiquitin structures at DNA damage sites, Science 316 (2007) 1198–1202. 17525341 10.1126/science.1139516 PMC2706583

[15] Mattiroli F, Vissers JH, van Dijk WJ, Ikpa P, Citterio E, Vermeulen W, Marteijn JA, Sixma TK, RNF168 ubiquitinates K13–15 on H2A/H2AX to drive DNA damage signaling, Cell 150 (2012) 1182–1195. 22980979 10.1016/j.cell.2012.08.005

[16] Bunting SF, Callen E, Wong N, Chen HT, Polato F, Gunn A, Bothmer A, Feldhahn N, Fernandez-Capetillo O, Cao L, Xu X, Deng CX, Finkel T, Nussenzweig M, Stark JM, Nussenzweig A, 53BP1 inhibits homologous recombination in Brca1-deficient cells by blocking resection of DNA breaks, Cell 141 (2010) 243–254. 20362325 10.1016/j.cell.2010.03.012 PMC2857570

[17] Setiaputra D, Durocher D, Shieldin - the protector of DNA ends, EMBO Rep 20 (2019). 10.15252/embr.201847560 PMC6501030 30948458

[18] Mirman Z, Lottersberger F, Takai H, Kibe T, Gong Y, Takai K, Bianchi A, Zimmermann M, Durocher D, de Lange T, 53BP1-RIF1-shieldin counteracts DSB resection through CST- and Polalpha-dependent fill-in, Nature 560 (2018) 112–116. 30022158 10.1038/s41586-018-0324-7 PMC6072559

[19] Fugmann SD, Lee AI, Shockett PE, Villey IJ, Schatz DG, The RAG proteins and V(D)J recombination: complexes, ends, and transposition, Annu Rev Immunol 18 (2000) 495–527. 10837067 10.1146/annurev.immunol.18.1.495

[20] Helmink BA, Sleckman BP, The Response to and Repair of RAG-Mediated DNA Double-Strand Breaks, Annu Rev Immunol (2012). 10.1146/annurev-immunol-030409-101320 PMC4038028 22224778

[21] Ahnesorg P, Smith P, Jackson SP, XLF interacts with the XRCC4-DNA ligase IV complex to promote DNA nonhomologous end-joining, Cell 124 (2006) 301–313. 16439205 10.1016/j.cell.2005.12.031

[22] Buck D, Malivert L, de Chasseval R, Barraud A, Fondaneche MC, Sanal O, Plebani A, Stephan JL, Hufnagel M, le Deist F, Fischer A, Durandy A, de Villartay JP, Revy P, Cernunnos, a novel nonhomologous end-joining factor, is mutated in human immunodeficiency with microcephaly, Cell 124 (2006) 287–299. 16439204 10.1016/j.cell.2005.12.030

[23] Li G, Alt FW, Cheng HL, Brush JW, Goff PH, Murphy MM, Franco S, Zhang Y, Zha S, Lymphocyte-specific compensation for XLF/cernunnos end-joining functions in V(D)J recombination, Mol Cell 31 (2008) 631–640. 18775323 10.1016/j.molcel.2008.07.017 PMC2630261

[24] Zha S, Alt FW, Cheng HL, Brush JW, Li G, Defective DNA repair and increased genomic instability in Cernunnos-XLF-deficient murine ES cells, Proc Natl Acad Sci U S A 104 (2007) 4518–4523. 17360556 10.1073/pnas.0611734104 PMC1838633

[25] Zha S, Guo C, Boboila C, Oksenych V, Cheng HL, Zhang Y, Wesemann DR, Yuen G, Patel H, Goff PH, Dubois RL, Alt FW, ATM damage response and XLF repair factor are functionally redundant in joining DNA breaks, Nature 469 (2011) 250–254. 21160472 10.1038/nature09604 PMC3058373

[26] Hung PJ, Chen BR, George R, Liberman C, Morales AJ, Colon-Ortiz P, Tyler JK, Sleckman BP, Bredemeyer AL, Deficiency of XLF and PAXX prevents DNA double-strand break repair by non-homologous end joining in lymphocytes, Cell Cycle 16 (2017) 286–295. 27830975 10.1080/15384101.2016.1253640 PMC5323033

[27] Oksenych V, Alt FW, Kumar V, Schwer B, Wesemann DR, Hansen E, Patel H, Su A, Guo C, Functional redundancy between repair factor XLF and damage response mediator 53BP1 in V(D)J recombination and DNA repair, Proc Natl Acad Sci U S A 109 (2012) 2455–2460. 22308489 10.1073/pnas.1121458109 PMC3289340

[28] Oksenych V, Kumar V, Liu X, Guo C, Schwer B, Zha S, Alt FW, Functional redundancy between the XLF and DNA-PKcs DNA repair factors in V(D)J recombination and nonhomologous DNA end joining, Proc Natl Acad Sci U S A 110 (2013) 2234–2239. 23345432 10.1073/pnas.1222573110 PMC3568359

[29] Liu X, Jiang W, Dubois RL, Yamamoto K, Wolner Z, Zha S, Overlapping functions between XLF repair protein and 53BP1 DNA damage response factor in end joining and lymphocyte development, Proc Natl Acad Sci U S A 109 (2012) 3903–3908. 22355127 10.1073/pnas.1120160109 PMC3309750

[30] Kumar V, Alt FW, Frock RL, PAXX and XLF DNA repair factors are functionally redundant in joining DNA breaks in a G1-arrested progenitor B-cell line, Proc Natl Acad Sci U S A 113 (2016) 10619–10624. 27601633 10.1073/pnas.1611882113 PMC5035843

[31] Abramowski V, Etienne O, Elsaid R, Yang J, Berland A, Kermasson L, Roch B, Musilli S, Moussu JP, Lipson-Ruffert K, Revy P, Cumano A, Boussin FD, de Villartay JP, PAXX and Xlf interplay revealed by impaired CNS development and immunodeficiency of double KO mice, Cell Death Differ 25 (2018) 444–452. 29077092 10.1038/cdd.2017.184 PMC5762856

[32] Tadi SK, Tellier-Lebegue C, Nemoz C, Drevet P, Audebert S, Roy S, Meek K, Charbonnier JB, Modesti M, PAXX Is an Accessory c-NHEJ Factor that Associates with Ku70 and Has Overlapping Functions with XLF, Cell Rep 17 (2016) 541–555. 27705800 10.1016/j.celrep.2016.09.026

[33] Hung PJ, Johnson B, Chen BR, Byrum AK, Bredemeyer AL, Yewdell WT, Johnson TE, Lee BJ, Deivasigamani S, Hindi I, Amatya P, Gross ML, Paull TT, Pisapia DJ, Chaudhuri J, Petrini JJH, Mosammaparast N, Amarasinghe GK, Zha S, Tyler JK, Sleckman BP, MRI Is a DNA Damage Response Adaptor during Classical Non-homologous End Joining, Mol Cell (2018). 10.1016/j.molcel.2018.06.018 PMC6083883 30017584

[34] Santos MA, Huen MS, Jankovic M, Chen HT, Lopez-Contreras AJ, Klein IA, Wong N, Barbancho JL, Fernandez-Capetillo O, Nussenzweig MC, Chen J, Nussenzweig A, Class switching and meiotic defects in mice lacking the E3 ubiquitin ligase RNF8, J Exp Med 207 (2010) 973–981. 20385748 10.1084/jem.20092308 PMC2867275

[35] Shull ER, Lee Y, Nakane H, Stracker TH, Zhao J, Russell HR, Petrini JH, McKinnon PJ, Differential DNA damage signaling accounts for distinct neural apoptotic responses in ATLD and NBS, Genes Dev 23 (2009) 171–180. 19171781 10.1101/gad.1746609 PMC2648541

[36] Bredemeyer AL, Sharma GG, Huang CY, Helmink BA, Walker LM, Khor KC, Nuskey B, Sullivan KE, Pandita TK, Bassing CH, Sleckman BP, ATM stabilizes DNA double-strand-break complexes during V(D)J recombination, Nature 442 (2006) 466–470. 16799570 10.1038/nature04866

[37] Helmink BA, Tubbs AT, Dorsett Y, Bednarski JJ, Walker LM, Feng Z, Sharma GG, McKinnon PJ, Zhang J, Bassing CH, Sleckman BP, H2AX prevents CtIP-mediated DNA end resection and aberrant repair in G1-phase lymphocytes, Nature 469 (2011) 245–249. 21160476 10.1038/nature09585 PMC3150591

[38] Hung PJ, Johnson B, Chen BR, Byrum AK, Bredemeyer AL, Yewdell WT, Johnson TE, Lee BJ, Deivasigamani S, Hindi I, Amatya P, Gross ML, Paull TT, Pisapia DJ, Chaudhuri J, Petrini JJH, Mosammaparast N, Amarasinghe GK, Zha S, Tyler JK, Sleckman BP, MRI Is a DNA Damage Response Adaptor during Classical Non-homologous End Joining, Mol Cell 71 (2018) 332–342, e338. 30017584 10.1016/j.molcel.2018.06.018 PMC6083883

[39] Forment JV, Walker RV, Jackson SP, A high-throughput, flow cytometry-based method to quantify DNA-end resection in mammalian cells, Cytometry A 81 (2012) 922–928. 22893507 10.1002/cyto.a.22155 PMC3601416

[40] Tubbs AT, Dorsett Y, Chan E, Helmink B, Lee BS, Hung P, George R, Bredemeyer AL, Mittal A, Pappu RV, Chowdhury D, Mosammaparast N, Krangel MS, Sleckman BP, KAP-1 Promotes Resection of Broken DNA Ends Not Protected by gamma-H2AX and 53BP1 in G1-Phase Lymphocytes, Mol Cell Biol (2014). 10.1128/MCB.00441-14 PMC4135573 24842905

[41] Dorsett Y, Zhou Y, Tubbs AT, Chen BR, Purman C, Lee BS, George R, Bredemeyer AL, Zhao JY, Sodergen E, Weinstock GM, Han ND, Reyes A, Oltz EM, Dorsett D, Misulovin Z, Payton JE, Sleckman BP, HCoDES reveals chromosomal DNA end structures with single-nucleotide resolution, Mol Cell 56 (2014) 808–818. 25435138 10.1016/j.molcel.2014.10.024 PMC4272619

[42] Symington LS, Gautier J, Double-strand break end resection and repair pathway choice, Annu Rev Genet 45 (2011) 247–271. 21910633 10.1146/annurev-genet-110410-132435

[43] Andres SN, Vergnes A, Ristic D, Wyman C, Modesti M, Junop M, A human XRCC4-XLF complex bridges DNA, Nucleic Acids Res 40 (2012) 1868–1878. 22287571 10.1093/nar/gks022 PMC3287209

[44] Andres SN, Modesti M, Tsai CJ, Chu G, Junop MS, Crystal structure of human XLF: a twist in nonhomologous DNA end-joining, Mol Cell 28 (2007) 1093–1101. 18158905 10.1016/j.molcel.2007.10.024

[45] Brouwer I, Sitters G, Candelli A, Heerema SJ, Heller I, de Melo AJ, Zhang H, Normanno D, Modesti M, Peterman EJ, Wuite GJ, Sliding sleeves of XRCC4-XLF bridge DNA and connect fragments of broken DNA, Nature 535 (2016) 566–569. 27437582 10.1038/nature18643

[46] Li Y, Chirgadze DY, Bolanos-Garcia VM, Sibanda BL, Davies OR, Ahnesorg P, Jackson SP, Blundell TL, Crystal structure of human XLF/Cernunnos reveals unexpected differences from XRCC4 with implications for NHEJ, EMBO J 27 (2008) 290–300. 18046455 10.1038/sj.emboj.7601942 PMC2104711

[47] Celeste A, Fernandez-Capetillo O, Kruhlak MJ, Pilch DR, Staudt DW, Lee A, Bonner RF, Bonner WM, Nussenzweig A, Histone H2AX phosphorylation is dispensable for the initial recognition of DNA breaks, Nat Cell Biol 5 (2003) 675–679. 12792649 10.1038/ncb1004

[48] Celeste A, Petersen S, Romanienko PJ, Fernandez-Capetillo O, Chen HT, Sedelnikova OA, Reina-San-Martin B, Coppola V, Meffre E, Difilippantonio MJ, Redon C, Pilch DR, Olaru A, Eckhaus M, Camerini-Otero RD, Tessarollo L, Livak F, Manova K, Bonner WM, Nussenzweig MC, Nussenzweig A, Genomic instability in mice lacking histone H2AX, Science 296 (2002) 922–927. 11934988 10.1126/science.1069398 PMC4721576

[49] Bothmer A, Robbiani DF, Di Virgilio M, Bunting SF, Klein IA, Feldhahn N, Barlow J, Chen HT, Bosque D, Callen E, Nussenzweig A, Nussenzweig MC, Regulation of DNA end joining, resection, and immunoglobulin class switch recombination by 53BP1, Mol Cell 42 (2011) 319–329. 21549309 10.1016/j.molcel.2011.03.019 PMC3142663

[50] Lukas J, Lukas C, Bartek J, More than just a focus: The chromatin response to DNA damage and its role in genome integrity maintenance, Nat Cell Biol 13 (2011) 1161–1169. 21968989 10.1038/ncb2344

[51] Averbeck NB, Ringel O, Herrlitz M, Jakob B, Durante M, Taucher-Scholz G, DNA end resection is needed for the repair of complex lesions in G1-phase human cells, Cell Cycle 13 (2014) 2509–2516. 25486192 10.4161/15384101.2015.941743 PMC4615131

[52] Biehs R, Steinlage M, Barton O, Juhasz S, Kunzel J, Spies J, Shibata A, Jeggo PA, Lobrich M, DNA Double-Strand Break Resection Occurs during Non-homologous End Joining in G1 but Is Distinct from Resection during Homologous Recombination, Mol Cell 65 (2017) 671–684, e675. 28132842 10.1016/j.molcel.2016.12.016 PMC5316416

