# Supplementary figures and images for: The RNF8 and RNF168 Ubiquitin Ligases Regulate Pro- and Anti-Resection Activities at Broken DNA Ends During Non-Homologous End Joining
Source: DNA Repair (Amst). Author manuscript; Available in PMC 2022 Oct 21. (PMC9586520; doi:10.1016/j.dnarep.2021.103217)

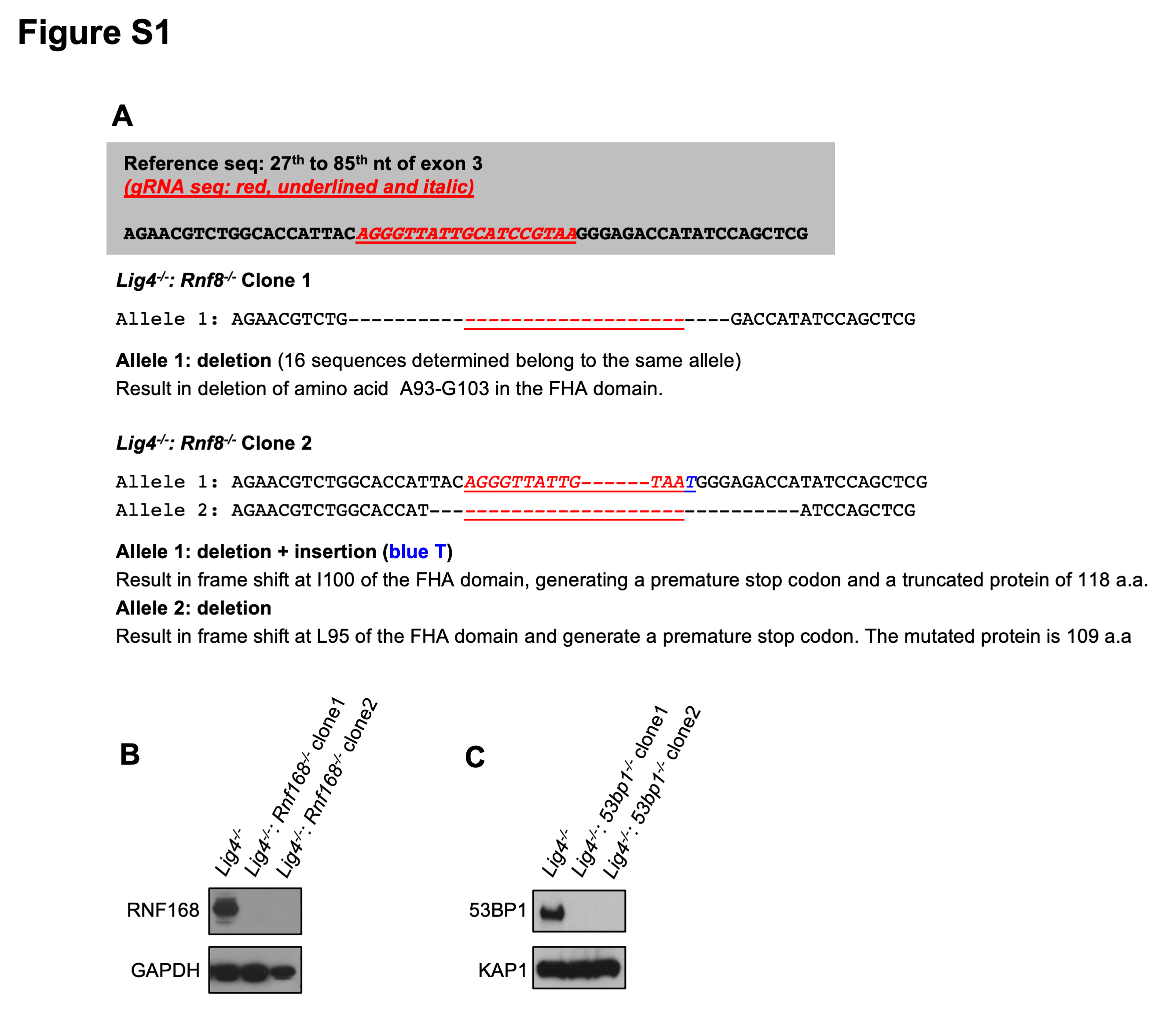

Supplement: Figure S1 [file NIHMS1748525-supplement-Figure_S1.jpg]

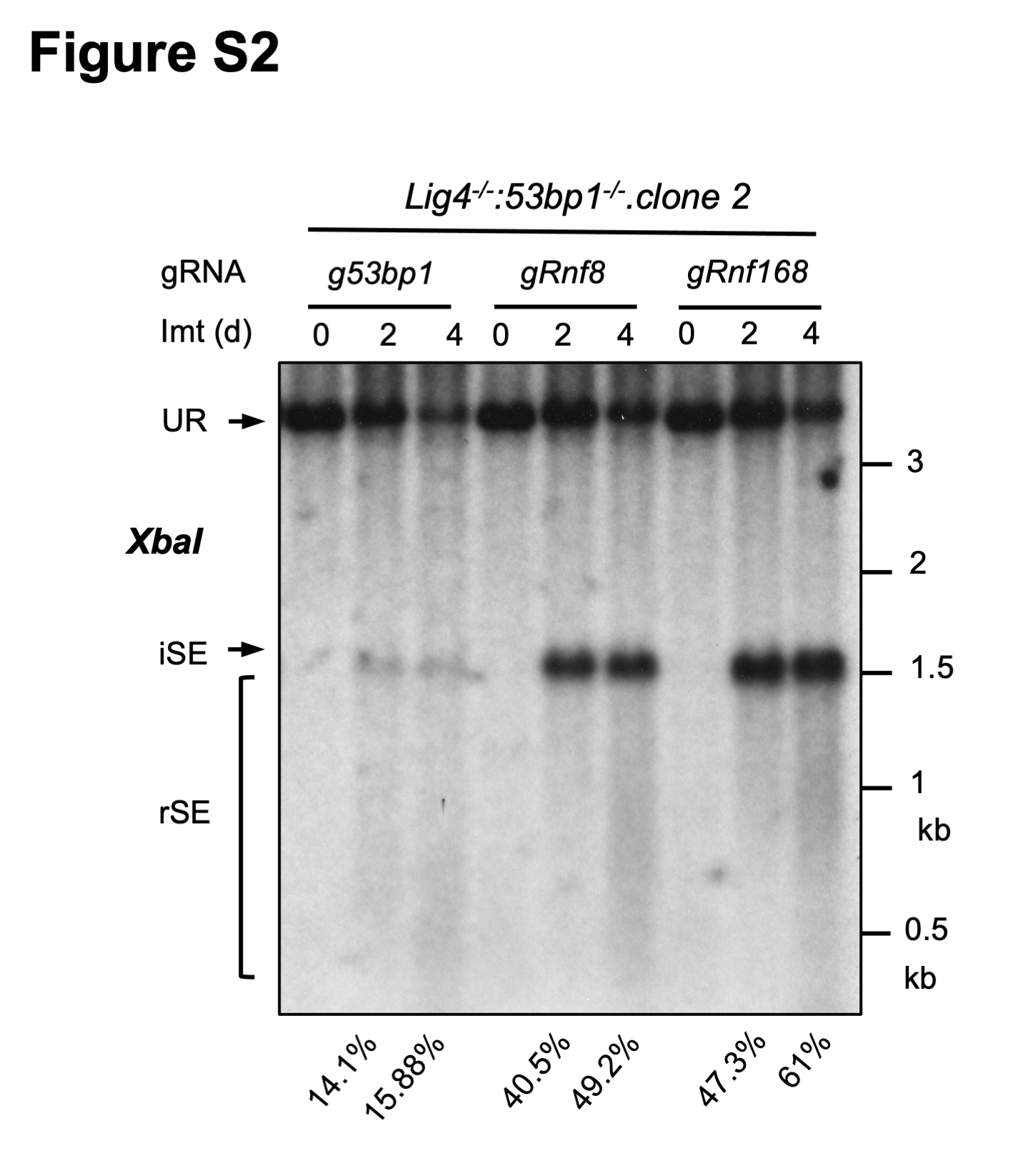

Supplement: Figure S2 [file NIHMS1748525-supplement-Figure_S2.jpg]
